# Preparation and Morphology Studies of Nano Zinc Oxide Obtained Using Native and Modified Chitosans

**DOI:** 10.3390/ma6094198

**Published:** 2013-09-18

**Authors:** Munusamy Thirumavalavan, Kai-Lin Huang, Jiunn-Fwu Lee

**Affiliations:** Graduate Institute of Environmental Engineering, National Central University, Chung-Li, Taoyuan County, 320, Taiwan; E-Mails: mtvala@yahoo.com (M.T.); kuroleo@msn.com (K.-L.H.)

**Keywords:** chitosans, modification, nano ZnO, SEM, BET

## Abstract

Nano zinc oxide (ZnO) with moderate surface area and high pore volume were prepared using a facile preparation method. Chitosan was utilized as both chelating and structure directing agent. The application of chitosans in this study suggested that even biowastes can be served in a productive manner economically. The surface modification of chitosan was carried out in order to increase the interaction between chitosan and zinc ions. The effect of sodium chloroacetate and isopropyl alcohol on the surface modification process was also explored. FT-IR (Fourier transform-infrared spectrometer) and TGA (Thermogravimetric analyses) analyses revealed that modified chitosans are more stable than those of unmodified chitosan. Among surface modified chitosans, CMC1 (1.5 M sodium chloroacetate and 75% isopropyl alcohol) showed enhanced surface properties. Freundlich adsorption isotherms as preliminary studies confirmed that modified chitosan showed enhanced interaction with zinc ions. The interaction of zinc salt with chitosans produced a zinc-chitosan polymer. This finally cleaved upon calcination to produce nano ZnO. The effects of different calcination temperatures indicated that 450 °C is the optimum calcination temperature to produce the nano ZnO with favored surface area (15.45 m^2^/g) and pore size (221.40 nm). SEM (Scanning electron microscope) and TEM (Transmission electron microscope) of ZnO indicated that uniform particle and shape distributions were obtained at low calcination temperature (450 °C).

## 1. Introduction

Zinc oxide (ZnO) has considerable customary attention due to its unique morphology and dimension-dependent optoelectronic properties [1]. It has special properties, such as high chemical activity, and novel optical, mechanical, electromagnetic, thermodynamic and electrodynamic properties, and displays a wide spectrum of applications, including gaseous sensors [2], fluorescent materials [3], photocatalysts [4], and additives in many industrial products [5]. Furthermore, ZnO is an environmentally friendly material, which is desirable especially for bio-applications, such as bio-imaging and cancer detection [6]. Several physical and chemical methods have been developed to obtain ZnO micro- and nanoparticles with different shapes. Various strategies such as chemical vapor deposition [7,8], electrochemical deposition [9,10,11], hydrothermal solution synthesis [12,13,14], and sol-gel processing [15] have been developed for the synthesis of ZnO nano materials. The sol-gel process and hydrothermal synthesis have proved to be relatively simple methods for synthesizing ZnO nanoparticles with a narrow size distribution and excellent crystallinity [16,17]. However, the organic materials utilized in the process make controlling of aggregation and large-scale production quite impossible. Various nanostructures in terms of shape and size have been found to hold novel applications depending upon on their morphologies and also the precursors [18,19,20,21]. Though various methods have been explored for synthesis of ZnO nanoparticles, we have developed a simple method, which involves the phenomenon of complex formation between metal ions and polysaccharides. Polysaccharides as stabilizing agents in this present task, met the requirement for the needs of cheap and renewable raw materials. Much of today’s research has further indicated that the capture of metal ions by forming complexes with polysaccharides has been used in the sequestration or removal of metal ions, solvent extraction, dyeing, catalysis, water treatment, and many other industrial processes [22]. It has also been realized [23], in recent years that the mechanism of complex formation of metals with polysaccharides is manifold and probably dominated by different processes taking place simultaneously, such as adsorption, ion-exchange, and chelation, under different conditions. Until now many methods have been developed to synthesize zinc oxide nanocrystals dispersed onto various supported materials. The common disadvantages of this method include the wide distribution of particle diameter, as well as the expensive raw materials. However, the use of metal-organic supramolecular compounds as precursors for the preparation of inorganic nanomaterials, such as ZnO, has not yet been thoroughly investigated.

This study attempts to take advantage of interaction between alkaline polysaccharides (both native and modified chitosans) and zinc metal salts to prepare nano ZnO. Generally, polysaccharides contain hydroxyl, and amino functional groups, which can form chelation with Zn metals by chemical adsorption. Finally calcination resulted into the cleavage of Zn-polysaccharides chelate to form nano ZnO. These polysaccharides in this study can effectively act as both chelating and structure directing agents. To enhance the degree of complexation of Zn metal and polysaccharides, necessary surface modification shave been carried out in this study. The effects of reaction temperature, concentration of solvents, and concentration of reactants were also studied during surface modification. The adsorption efficiency of these chitosans towards zinc metal was also explored in this study using Freundlich adsorption isotherms. The cheap, high stability, low toxicity, and environmentally friendly features of these polysaccharides along with simple and convenient technology have enabled to obtain nano ZnO in this present investigation.

The objective of this investigation was to emphasize the significant application of low cost biowastes (chitosans) for the effective production of nanomaterials and also to elucidate the formation of coordinate linkages and the surface microstructure when Zn^2+^ ion complexes with native and surface modified chitosan under specific conditions. In the present work, the effects of the different reaction conditions on particle size, and morphology were studied and the surface properties of various nano ZnO obtained from different chitosans were compared.

## 2. Experimental

### 2.1. Chemicals

Deionized distilled water was used to prepare all solutions. Standard metal ion solution, Zinc nitrate hexahydrate [Zn(NO_3_)_2_·6H_2_O] was commercially obtained from J.T. Baker. Low molecular weight chitosan (molecular weight: 140 kDa) was obtained commercially from Aldrich. All the chemicals and reagents used in this study were of analytical grade and used without any further purification.

### 2.2. Chemical Modification of Chitosan

Chemically-modified chitosan was prepared according to the modified methods [24,25] by alkalization and etherification. The reaction parameters, such as concentrations of isopropyl alcohol and sodium chloroacetate, were varied to obtain a variety of modified chitosans. Ten grams (10 g) of chitosan powder was dispersed in 100 mL of different concentration ratio (75% and 50%) of isopropyl alcohol and stirred at 28 °C for 30 min. A solution of 25 mL 10 M sodium hydroxide was added in five equal portions over 25 min (at an interval of 5 min) under stirring and continued to stir for additional 30 min. Following this, different concentrations (1.5, 2.25 and 3 M) of sodium chloroacetate dissolved in 45 mL 10 N sodium hydroxide and small amount of water were added to the mixture. Following this, the reaction mixture was stirred for 3 h at 60 °C. The resultant solution was filtered and washed with ethanol for three times, then dried in an oven at 60 °C for one day to get modified chitosan. Chitosan was referred to as CTS. Chemically modified chitosans prepared using 1.5, 2.25, and 3 M sodium chloroacetate with 75% isopropyl alcohol were referred to as CMC1, CMC2, and CMC3 respectively. Chemically modified chitosans prepared using 1.5, 2.25, and 3 M sodium chloroacetate with 50% isopropyl alcohol were referred to as CMC4, CMC5, and CMC6 respectively.

### 2.3. Adsorption Studies

In order to understand the affinity of native and modified chitosan towards zinc metal ions, adsorption experiment was carried out as a primary study and fitted with Freundlich isotherm model.

The adsorption of Zn^2+^ was investigated in batch equilibrium experiments using zinc nitrate solution. The experiments were performed in 25 mL centrifuge bottle by stirring 25 mL zinc ion solution and 0.1 g of the adsorbents (native and surface modified chitosan) at 130 rpm in a Lab-line orbit environ shaker for 24 h. The temperature was maintained at 28 °C. The adsorption of metal ions from the aqueous solutions was studied. After the desired reaction period, the aqueous phases were separated from the materials by centrifugation at 4500 rpm for 5 min and the concentration of metal ions was measured using an AA-400 atomic absorption spectrophotometer (AAS, Varian, Inc., Palo Alto, CA, USA).

### 2.4. Synthesis of ZnO Using Native and Surface Modified Chitosan

Three gram of zinc nitrate hexahydrate [Zn(NO_3_)_2_·6H_2_O] was dissolved in 100 mL water in a standard flask. To this zinc nitrate aqueous solution, 3 g of native and surface modified chitosan was added respectively and the reaction mixture was stirred constantly at 28 °C for 6 h. Following this, the reaction mixture was filtered and the solid collected was dried in an oven at 50 °C to obtain zinc-chitosan organic polymers. Finally this zinc-chitosan organic polymer was calcined at three different temperatures, namely 450, 650, and 860 °C, to obtain ZnO nanostructures such as ZnO-CTS (from CTS), ZnO-CMC1 (from CMC1), ZnO-CMC2 (from CMC2), ZnO-CMC3 (from CMC3), ZnO-CMC4 (from CMC4), ZnO-CMC5 (from CMC5), and ZnO-CMC6 (from CMC6) with various morphologies. ZnO samples obtained from various systems were referred as shown in Table 1.

**Table 1:** ZnO prepared from various systems.

**Precursors**	**ZnO samples prepared at various calcination temperatures of Zn-chitosan polymers**		
	450 °C	650 °C	850 °C
CTS	ZnO-CTS-450	ZnO-CTS-650	ZnO-CTS-850
CMC1	ZnO-CMC1-450	ZnO-CMC1-650	ZnO-CMC1-850
CMC2	ZnO-CMC2-450	ZnO-CMC2-650	ZnO-CMC2-850
CMC3	ZnO-CMC3-450	ZnO-CMC3-650	ZnO-CMC3-850
CMC4	ZnO-CMC4-450	ZnO-CMC4-650	ZnO-CMC4-850
CMC5	ZnO-CMC5-450	ZnO-CMC5-650	ZnO-CMC5-850
CMC6	ZnO-CMC6-450	ZnO-CMC6-650	ZnO-CMC6-850

### 2.5. Characterization Techniques

The average pore diameter and specific surface area [BET (Brunauer–Emmett–Teller) surface and pore volume] were measured on a Quantochrome NOVA 1000 (Boynton Beach, FL, USA). XRD patterns were obtained at room temperature using a Bruker KAPPA APEX II instrument (Billerica, MA, USA). Scanning electron microscope SEM study was carried out on an HITACHI-S-800, field emission scanning electron microscope. TEM study was carried out on a transmission electron microscopy (JEM-2010type). FT-IR spectra were obtained on a Neclit 6700 model, FTIR. TGA was performed with Universal V4.4A (TA Instruments, New Castle, DE, USA).

## 3. Results and Discussion

### 3.1. Characterization of Native and Surface Modified Chitosans

**Figure 1:**
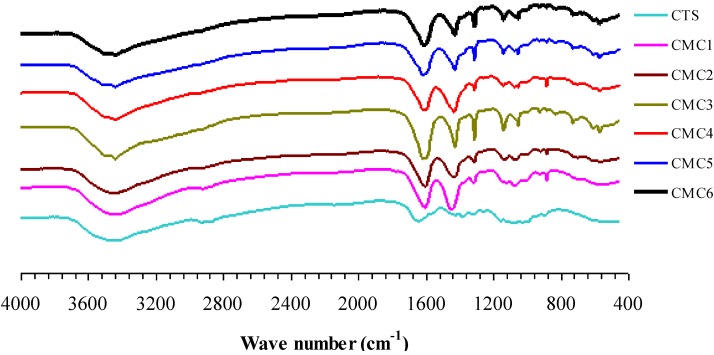
FT-IR spectra of native and modified chitosans.

The functional groups present in chitosan and modified chitosan were identified using FT-IR technique. Figure 1 shows the FT-IR spectra of native and surface modified chitosans. The peak [26] around 3480 cm^−1^, is due to ν(O–H), the peak at 2800 to 3000 cm^−1^ is due to ν(CH_3_, CH_2_, CH, and NH), the peak at 1630 to 1650 cm^−1^ is due to ν(C=O), the peak around 1400 to 1500 cm^−1^ is due to ν(CO) deformation from alcoholic and phenolic and symmetric ν(COO) and the peak at 1050 to 1300 cm^−1^ is due to ν(C–O) of chitosan. It can be seen from Figure 1 that the broadness of the peak around 3480 cm^−1^ is gradually diminished upon surface modification, which indicates the decrease of water and enhancement of carboxylic functional groups in chitosan. In addition, significant increase of C=O peak intensity around 1630 to 1650 cm^−1^ confirms the higher degree of carboxylic group of modified chitosan. It is connoted that the intensity [27] of ν(O–H) and ν(C=O) was significantly affected by the factors such as concentrations of sodium chloroacetate and isopropyl alcohol. It is observed when concentration of sodium chloroacetate increased the intensity and peak width of FT-IR spectra of modified chitosan around 1630 to 1650 cm^−1^ also increased significantly. Despite the increase in ν(C=O), the intensity of peak around 1400 to 1500 cm^−1^also increased definitely. This clearly evidenced that, at higher concentration of sodium chloroacetate, the deformation of C=O enhanced and the free COO^−^ on chitosan surface is diminished.

**Figure 2:**
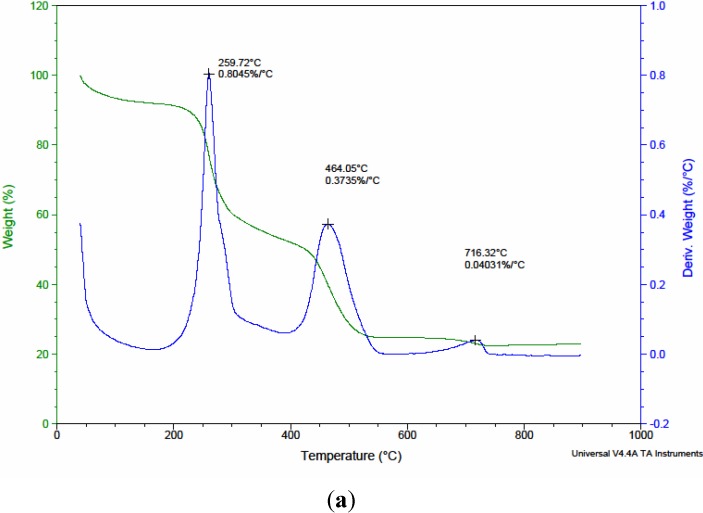

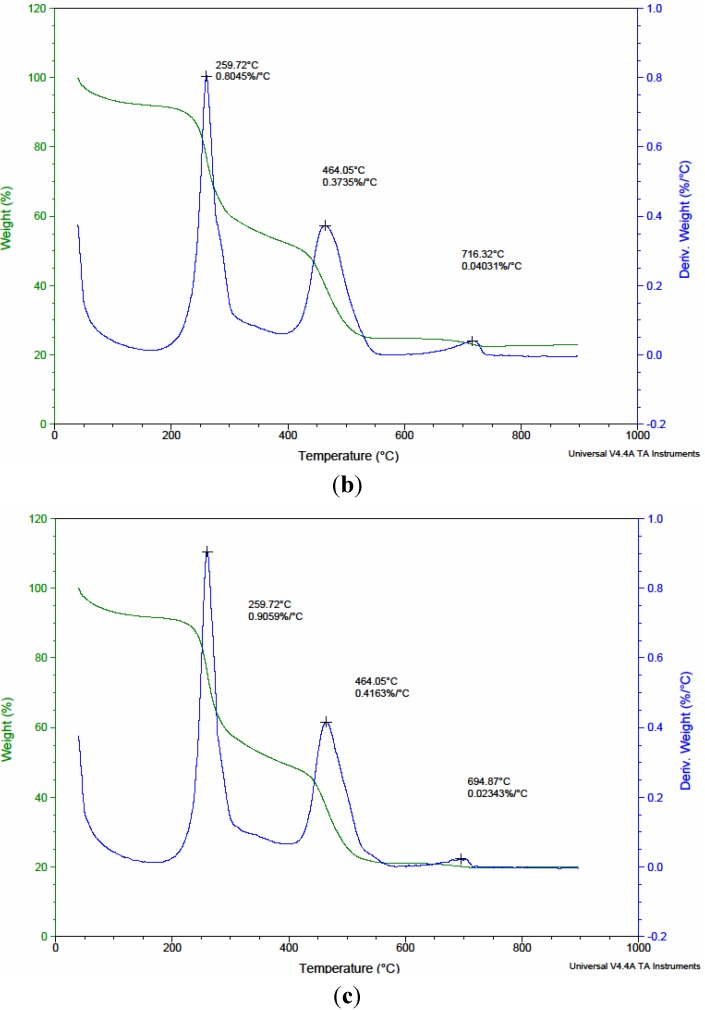
TGA and DTA curves for zinc-chitosans organic polymers using (**a**) CTS; (**b**) CMC1; and (**c**) CMC4.

There will be no significant change in the NH_2_ peak upon coordination with metal ions and, moreover, making the differentiation is also not always possible. It is expected that the carboxyl groups of the chitosan can interact with zinc metal to form chitosan-zinc complexes. In general, the un-ionized and uncoordinated carboxylic acid (C=O) stretching band occurs at 1700–1750 cm^−^^1^, whereas the ionized and coordinated stretching band occurs at 1650–1590 cm^−1^. In this study, no absorption band in the region of 1700–1750 cm^−^^1^ was observed, indicating that the carboxyl groups have a high degree of an ionic characteristic. The ionized carbonyl frequencies in chelating samples suggest that the chelation of chitosan with Zn^2+^occurred in the ionized moiety of the carboxyl groups. Thermogravimetric analyses of the zinc-chitosans organic polymers using native and surface modified chitosan were conducted from room temperature (RT) to 900 °C. The TGA and DTA curves for a typical precursor are shown in Figure 2. The weight loss curves of the native chitosan in the range 200–450 °C are associated with the decomposition of chitosan [28]. The corresponding weight loss curves of the modified chitosans, in the range 200–700 °C, imply the multiple weight loss events of modified chitosan. The residual weight (remaining) of chitosans during TGA analysis is tabulated in Table 2. From Table 2, it is seen that the residual weight of modified chitosans after various pyrolysis temperatures is more than that of native chitosan indicating that the modified chitosans are more stable [25] than those of native chitosan. Among all, CMC1 has more residual weight after TGA and thus considered to be the most stable chitosan in this study. A systematic correlation study carried out between two different concentrations (75% and 50%) of isopropyl alcohol revealed that for the same system with 75% isopropyl alcohol, the corresponding weight loss is lower than that of 50% isopropyl alcohol. This is due to the fact that the water puffing extent of 50% isopropyl system is greater than that of 75% isopropyl system. Thus the larger weight loss is accompanied with 50% isopropyl alcohol. Table 2, clearly conveyed that there is no obvious correlation between different concentrations of sodium chloroacetate.

**Table 2:** Residual weight of zinc-chitosans organic polymers during TGA after different pyrolysis temperatures.

Precursors	Residual weight (%) of precursors after pyrolysis		
	450 °C	650 °C	850 °C
CTS	34.05	11.45	11.40
CMC1	53.11	36.80	32.02
CMC2	48.94	29.61	25.10
CMC3	52.92	34.05	30.89
CMC4	31.66	23.92	23.00
CMC5	42.52	20.87	19.99
CMC6	41.56	15.49	15.42

### 3.2. Zn^2+^ Adsorption Studies

**Figure 3:**
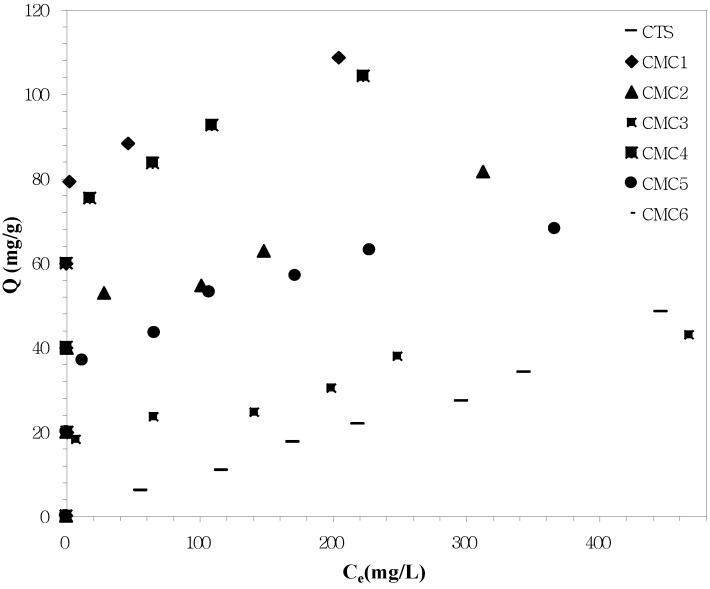
Adsorption of Zn^2+^ ions by native and surface modified chitosans.

Prior to the preparation of nano ZnO, it is important to have an idea about the affinity of both native and surface modified chitosan towards zinc metal ions. Hence, we have carried out adsorption of Zn^2+^ in solution as a preliminary study using all chitosans as adsorbents. The adsorption studies carried out in this work suggested that as compared to another models, Freundlich isotherms showed the best fitting value for the kinetics of Zn^2+^ on chitosans and the adsorption process is chemical adsorption. Figure 3 shows the Freundlich adsorption isotherms of different chitosans for Zn^2+^ ion, and Table 3 includes the Freundlich isotherm parameters of different systems. The experimental results clearly revealed that unmodified chitosan generally showed lower affinity towards Zn^2+^ than modified chitosan [29]. Thus, it is corroborated, that due to surface modification, the affinity of chitosans towards Zn^2+^ is enhanced owing to the presence of both COOH and NH_2_ groups. Among all, CMC1 showed the enhanced Zn^2+^ adsorption with increased K (capacity of the adsorbent for the adsorbate, mg/g) and n (constant) values. The order of adsorption capacity of the chitosans can be arranged as CMC1> CMC4 > CMC2 > CMC5 > CMC6 > CTS > CMC3. Based on this, it is observed that both the concentrations of sodium chloroacetate and isopropyl alcohol are the two major factors that control the adsorption process. The fundamental results indicated that smaller the sodium chloroacetate concentration and higher the isopropyl alcohol concentration, the greater the Zn^2+^ affinity. If sodium chloroacetate concentration is increased, instead of replacement of OH by COOH, the NH_2_ on chitosan surface is replaced by NHCH_3_COONa and, thus, the free NH_2_ sites are decreased. Hence, the adsorption of Zn^2+^ is diminished if the sodium chloroacetate concentration is increased.

However, higher concentration of isopropyl alcohol facilitated the adsorption of Zn^2+^ ions due to smaller water puffing extent during surface modification. When the concentration of isopropyl alcohol is increased, it prevents the aggregation of the particles due to water swelling effect and makes the system more homogeneous. In addition, higher concentration of isopropyl alcohol prevents the hydrolysis reaction during surface modification by increasing the utilization of sodium chloroacetate.

**Table 3:** Freundlich isotherm parameters for adsorption of Zn^2+^ by different chitosans.

Precursors	*R*^2^	K (capacity of the adsorbent for the adsorbate, mg/g)	*n* (constant)
CTS	0.9918	9.85	1.0040
CMC1	0.9547	69.78	12.9032
CMC2	0.9333	32.36	7.0373
CMC3	0.9438	5.29	2.9551
CMC4	0.9556	51.17	7.8612
CMC5	0.9828	24.00	5.9277
CMC6	0.9522	20.20	6.7843

### 3.3. Characterization of ZnO

**Figure 4:**
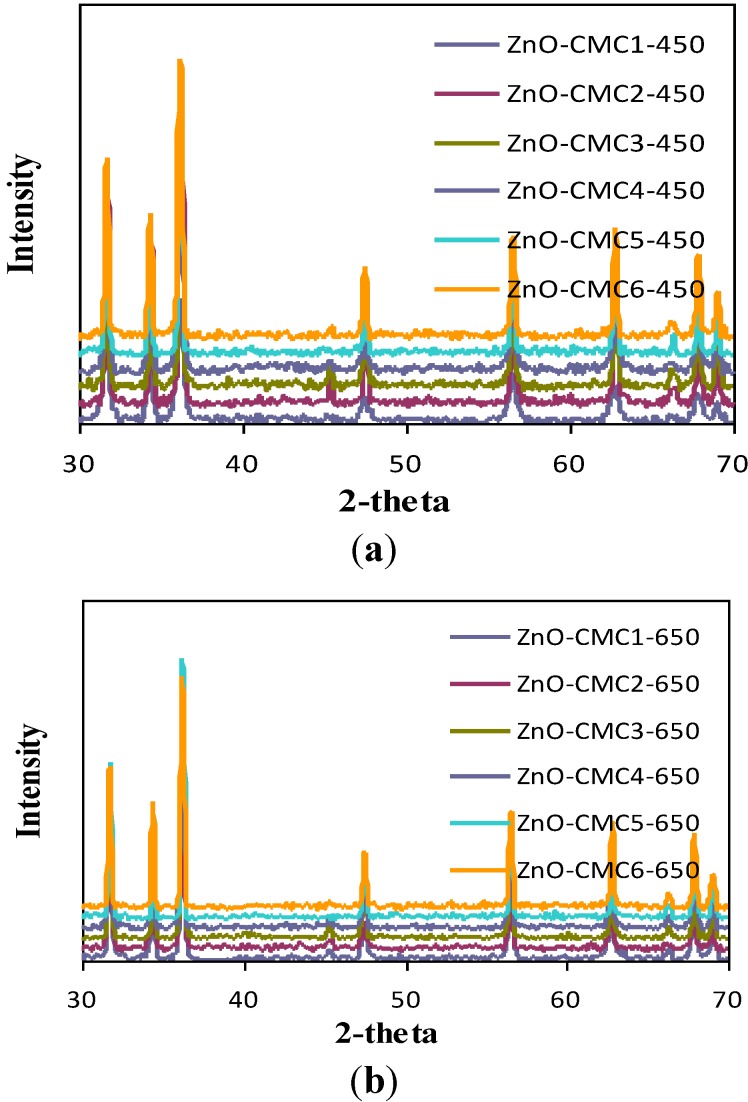

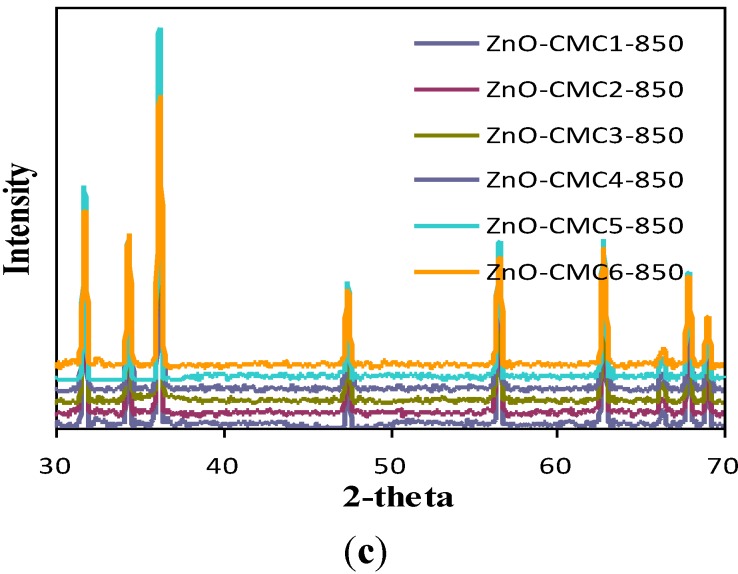
XRD patterns of various ZnO samples.

**Figure 5:**
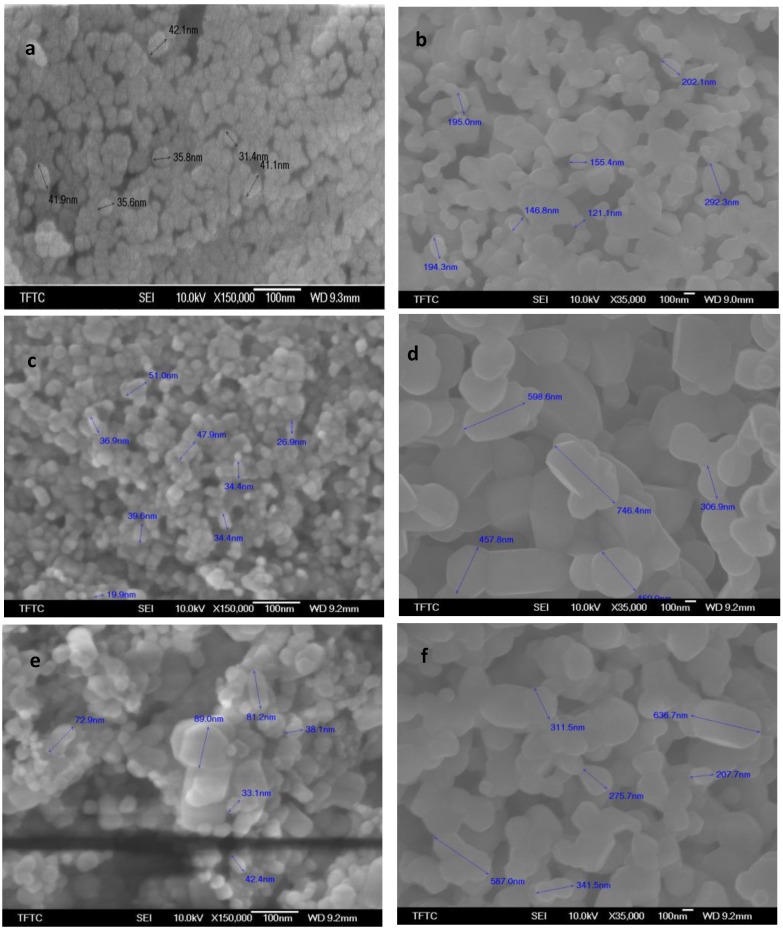
SEM of (**a**) ZnO-CTS-450; (**b**) ZnO-CTS-850; (**c**) ZnO-CMC1-450; (**d**) ZnO-CMC1-850; (**e**) ZnO-CMC4-450; and (**f**) ZnO-CMC4-850.

The facile preparation of nano ZnO was obtained from the cleavage of various zinc-polysacchraide polymers formed from chitosans and zinc nitrate solution. ZnO was characterized using XRD, SEM, TEM and BET analyses. The XRD patterns of nano ZnO at different calcination temperatures using various chitosans as chelating agents are given in Figure 4. The diffraction peaks indicate the nanocrystalline nature and identical to the hexagonal phase with Wurtzite structure. The peaks at angles (2θ) around 31°, 35°, 37°, 48°, 56°, 62°, 68°, and 69° correspond to the reflection from 100, 002, 101,102, 110, 103, 200 to 112 crystal planes, respectively [30]. The ZnO crystal structure seemed to be unchanged upon calcination at high temperatures but that the crystal size continually increased [31]. From Figure 4 it is connoted that, when the calcination temperature of zinc-chitosan polymer is low (450 °C), the XRD patterns of ZnO obtained from various chitosans are well separated from each other (*i.e.*, large separation in terms of intensity) where as in the case of 650 and 850 °C, they are almost close to each other (*i.e.*, less separation in terms of intensity). The SEM of ZnO nanocrystalline particles obtained by various systems is shown in Figure 5. As seen in Figure 5, almost single phase primary particle in spherical shape with uniform distribution was obtained at low calcination temperature (450 °C) of zinc-chitosan polymer where as in the case of high calcination temperatures particles with various shapes and sizes were obtained. Thus, at high calcination temperature of zinc-chitosan polymer, disordered structural distribution of ZnO was witnessed and sometimes even structural damage.

The particle size of various ZnO obtained is given in Table 4. From Table 4, it is clear that as the calcination temperature of zinc-chitosan polymer increased, the particle size of obtained ZnO also increased. This clearly explained that nano ZnO with same crystal type but different particle size can be obtained by varying the calcination temperature [32]. The surface areas are found to be decreased while crystal sizes of the nanoparticles held a certain degree of agglomeration as calcination temperatures gradually increased [31]. Thus, at higher calcination temperatures, the agglomeration of ZnO nanoparticles became extremely serious and hence the particle size increased. This may be explained as sample effects results from two main effects: (i) the particle-size broadening which results from the finite extent and particular morphology of the coherently diffracting domains within the grains; and (ii) the microstrain broadening, which results from local variations of the d-spacing produced by nonuniform crystalline stresses.

**Table 4:** Particle size of ZnO prepared at different calcination temperatures of Zn-chitosan polymers.

ZnO samples	Particle size of ZnO (nm)		
	450 °C	650 °C	850 °C
ZnO-CTS	31–42	74–193	121–292
ZnO-CMC1	19–54	91–220	306–746
ZnO-CMC2	59–107	72–198	300–835
ZnO-CMC3	43–124	135–519	580–584
ZnO-CMC4	26–69	65–683	207–636
ZnO-CMC5	32–70	67–380	226–1069
ZnO-CMC6	33.89	96–211	237–345

**Figure 6:**
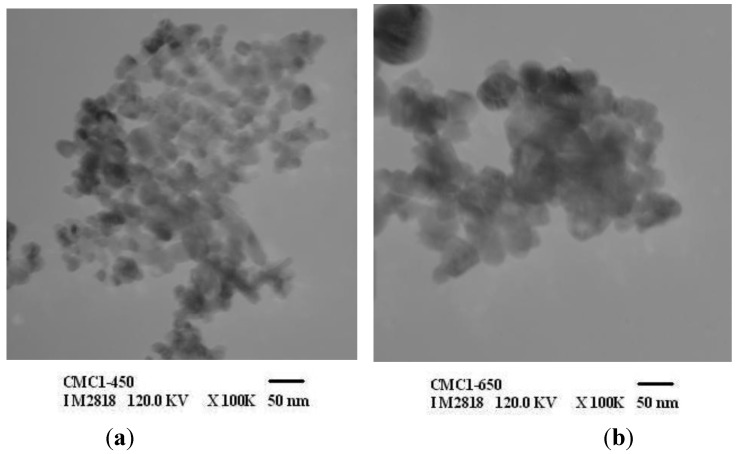

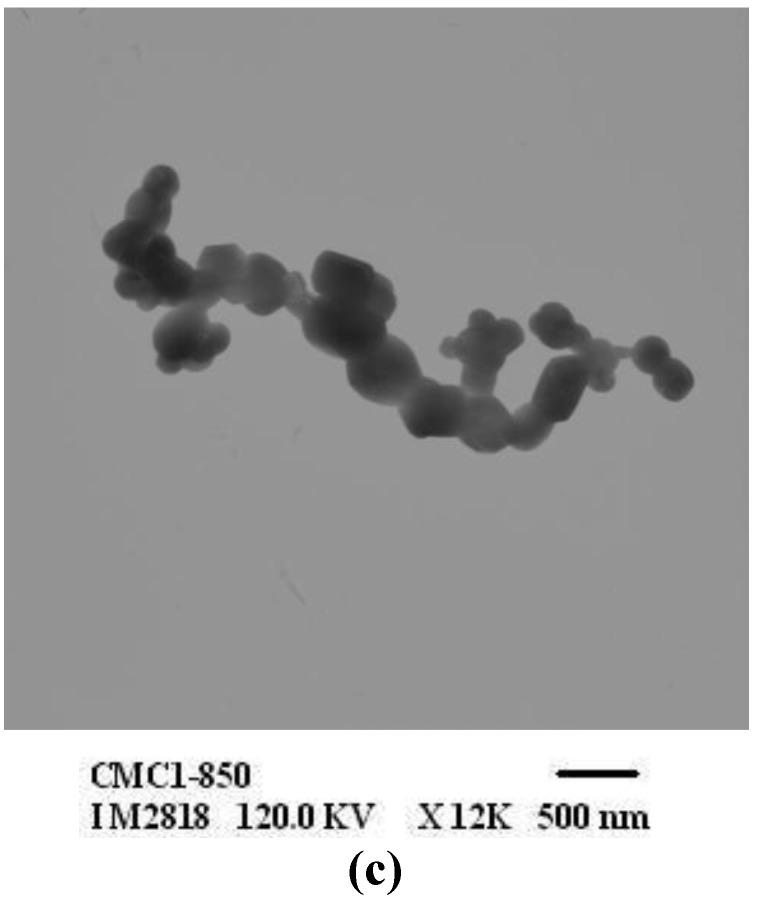
TEM of (**a**) ZnO-CMC1-450; (**b**) ZnO-CMC1-650; and (**c**) ZnO-CMC1-850.

The size and morphology of ZnO particles analyzed by TEM is represented in Figure 6. This also revealed the uniform size and shape distribution of nano ZnO obtained at low calcination temperatures of zinc-chitosan polymer.

The hydration ratio [33] can also affect the hydrolysis and condensation rate of the Zn precursor in solution, as evidenced in the case of 75% and 50% isopropyl alcohol systems, by which ZnO can nucleate and grow into distinct particle morphology. The experimental data showed that varying the synthetic conditions, significantly affected the specific surface area and pore size distribution of obtained ZnO as shown in Table 5.

**Table 5:** Surface area and pore size of different ZnO samples prepared.

Samples	BET surface area (m^2^/g)	Average pore diameter (nm)
ZnO-CTS-450	23.7664	9.70
ZnO-CMC1-450	15.4485	221.40
ZnO-CMC2-450	4.7763	106.29
ZnO-CMC3-450	4.5767	161.62
ZnO-CMC4-450	14.1519	212.03
ZnO-CMC5-450	6.0641	119.69
ZnO-CMC6-450	6.0966	131.10
ZnO-CTS-650	11.9273	6.97
ZnO-CMC1-650	5.8813	160.49
ZnO-CMC2-650	2.9608	108.93
ZnO-CMC5-650	2.3485	8.96
ZnO-CMC6-650	3.1625	10.59

As discussed earlier, unmodified chitosan showed low affinity for Zn^2+^ ions which necessarily initiated the surface modification of chitosan for the enhanced affinity for Zn^2+^ ions. Surface modification of chitosans perhaps eases the formation of zinc-chitosan polymers. However, quite interestingly, nano ZnO obtained from cleavage of zinc-unmodified chitosan polymer possessed a larger surface area and an exceptionally smaller pore size. However, still surface modification is indispensable for the effective interaction of chitosans with zinc ions. Among surface modified chitosans, ZnO obtained from cleavage of zinc-CMC1 had a significantly higher surface area and larger pore size than those of others. The findings of this work can be concluded as different parameters affect the efficiency of zinc-modified chitosan polymers to produce nano ZnO and among which 1.5 M sodium chloroacetate, 75% isopropyl alcohol and calcination at 450 °C are the optimized parameters to obtain nano ZnO with enhanced surface area.

## 4. Conclusions

A facile preparation of nano ZnO was reported in this work. Native and surface modified chitosans were used as both chelating and structure directing agents. Upon surface modification (prior to ZnO synthesis), sodium chloroacetate and isopropyl alcohol were the major factors that controlled the stability and surface property of modified chitosans. These modified and unmodified chitosans were characterized using FT-IR and TGA analyses. In order to understand the interaction of these polysachharides with zinc ions, adsorption experiments were carried out as preliminary studies and Freundlich adsorption isotherms were reported. It was observed that surface modified chitosans showed enhanced adsorption towards zinc ions. Reaction of these modified and unmodified chitosans with zinc salt was carried out to produce zinc-organic polymer. Finally upon calcination, the cleavage of these polymer produced nano ZnO. The obtained ZnO was characterized using XRD, FT-IR, SEM, TEM, and BET analysis. The effect of different calcination temperature was also studied. This study indeed suggested that a cost effective and viable technology for effective preparation of nano ZnO could be developed using easily available, familiar, and eco-friendly chitosans.
